# Body shape and size in 6-year old children: assessment by three-dimensional photonic scanning

**DOI:** 10.1038/ijo.2016.30

**Published:** 2016-04-05

**Authors:** L P Santos, K K Ong, F Day, J C K Wells, A Matijasevich, I S Santos, C G Victora, A J D Barros

**Affiliations:** 1 Postgraduate Program in Epidemiology, Federal University of Pelotas, Pelotas, Brazil; 2 Medical Research Council (MRC) Epidemiology Unit, Institute of Metabolic Science, Cambridge, UK; 3 Childhood Nutrition Research Centre, UCL Institute of Child Health, London, UK; 4 Department of Preventive Medicine, School of Medicine, University of São Paulo, São Paulo, Brazil

## Abstract

**Background:**

Body shape and size are typically described using measures such as body mass index (BMI) and waist circumference, which predict disease risks in adults. However, this approach may underestimate the true variability in childhood body shape and size.

**Objective:**

To use a comprehensive three-dimensional photonic scan approach to describe variation in childhood body shape and size.

**Subjects/Methods:**

At age 6 years, 3350 children from the population-based 2004 Pelotas birth cohort study were assessed by three-dimensional photonic scanner, traditional anthropometry and dual X-ray absorptiometry. Principal component analysis (PCA) was performed on height and 24 photonic scan variables (circumferences, lengths/widths, volumes and surface areas).

**Results:**

PCA identified four independent components of children's body shape and size, which we termed: Corpulence, Central:peripheral ratio, Height and arm lengths, and Shoulder diameter. Corpulence showed strong correlations with traditional anthropometric and body composition measures (*r*>0.90 with weight, BMI, waist circumference and fat mass; *r*>0.70 with height, lean mass and bone mass); in contrast, the other three components showed weak or moderate correlations with those measures (all *r*<0.45). There was no sex difference in Corpulence, but boys had higher Central:peripheral ratio, Height and arm lengths and Shoulder diameter values than girls. Furthermore, children with low birth weight had lower Corpulence and Height and arm lengths but higher Central:peripheral ratio and Shoulder diameter than other children. Children from high socio-economic position (SEP) families had higher Corpulence and Height and arm lengths than other children. Finally, white children had higher Corpulence and Central:peripheral ratio than mixed or black children.

**Conclusions:**

Comprehensive assessment by three-dimensional photonic scanning identified components of childhood body shape and size not captured by traditional anthropometry or body composition measures. Differences in these novel components by sex, birth weight, SEP and skin colour may indicate their potential relevance to disease risks.

## Introduction

Body shape is frequently assessed in epidemiological studies in adults to predict risks of mortality and other adverse health outcomes.^1, 2^ In addition to overall body weight-for-height (body mass index (BMI)), those studies often also assess measures of central body shape (for example, waist and hip circumferences, waist–hip ratio and regional fat mass distribution), which reflect the characteristic sexual dimorphism in body shape and body fat distribution.^3, 4, 5^ Men have relatively more central fat (‘android' distribution) while women have relatively more peripheral fat (‘gynoid'), a difference which is explained by sex hormone actions.^6^

In contrast to convincing evidence in adults that waist circumference and BMI combine synergistically to predict later disease risks,^1, 7^ the added value of estimating central body shape in children is less clear. Four studies have assessed the contribution of waist circumference to cardiovascular disease risk factors in children; only one (a study of 154 overweight or obese girls aged 5–16 years) reported a positive association between waist circumference and fasting insulin resistance that was independent of BMI.^8^ The other three studies were population based (sample sizes were 436, 5235 and 7589 children aged 7–12 years) and reported no additional contribution of waist circumference beyond BMI alone.^9, 10, 11^ Furthermore, measures of BMI and central body shape (such as waist circumference) show much higher inter-correlation in children than in adults, and therefore regression models that include both parameters are often affected by colinearity.^12, 13^ Finally, restriction of assessment of body shape to disease markers in adults may underestimate the wider variations in childhood body shape and size.

In recent years, three-dimensional (3-D) photonic scanning has been used in several studies to assess body shape and size in adults.^14, 15, 16^ The advantage of this approach is its ability to rapidly capture many diverse measurements (circumferences, widths, lengths) at different sites.^17^ Nevertheless, only one study has assessed body shape in children using 3-D photonic scanning.^18^ We used this method to describe variation in body shape and size in children and to assess how well these dimensions are captured by traditional anthropometric and body composition measurements.

## Methods

### Subjects

Pelotas is a city located in Southern Brazil, with a population of 330 000 inhabitants according to the 2010 Brazilian Demographic Census.^19^ The third Pelotas birth cohort study targeted all births in the city in 2004 and successfully recruited 99.2% of all babies born to mothers living in the urban area. In the first 24 h after birth, mothers were interviewed and newborns assessed by trained field workers at the maternity hospital. A questionnaire was applied by trained interviewers to collect information about the child, family, mother, pregnancy and birth. At ages 3, 12, 24 and 48 months and 6 years, the cohort children were followed up with retention rates of 95.7, 94.2, 93.4, 91.8 and 90.2%, respectively. Information about anthropometric variables, nutritional status, child development and socio-economic position (SEP) were collected in all follow-ups. Details of the perinatal and subsequent assessments are reported previously.^20, 21^ The study protocol was approved by the Research Ethics Committee of the Federal University of Pelotas, affiliated with the Brazilian Medical Council, and confidentiality of information was warranted.

### Anthropometric, body composition and photonic scan measures at age 6 years

The fifth follow-up of the cohort, at mean age 6.8 years (min. 5.8–max. 7.6 years), occurred between October 2010 and August 2011, required a visit to the study clinic and followed up 3722 children. The examination included traditional anthropometry (weight, height, sitting height and waist circumference), body composition assessment (whole-body dual X-ray absorptiometry (DXA, GE Lunar Prodigy densitometer, UK) and air-displacement plethysmography, Cosmed, Rome, Italy) and questionnaire assessment of lifestyle and health status.^21^ Standard anthropometry and body composition measures were collected by trained field workers. Quality-control measures included the use of standardized procedures and calibration of equipment (DXA and air-displacement plethysmography, Cosmed) before data collection, according to the manufacturer's specifications. The field workers who carried out anthropometric measures were standardized based on Habitch criteria.^22^ Weight was measured to the nearest 0.1 g on a calibrated and high precision scale (model BWB-627-A, Tanita Corporation, Tokyo, Japan, modified by Life Measurement, Inc., Concord, CA, USA); standing and sitting height were measured to the nearest 0.1 cm on a calibrated stadiometer (Harpenden, Holtain, Crymych, UK); waist circumference was tape-measured at the widest point between the lowest rib and the crest iliac. For all measures, participants were barefoot and wearing tight-fitting gym suits provided by the study.

Body shape and size were assessed by the TC2 Three-Dimensional Photonic Scanner (TC2, Cary, NC, USA; www.tc2.com). The equipment was originally designed for the clothing industry but has also been used in clinical and epidemiological research settings.^5, 14, 15^ All body scans were performed using the scanner's Body Scan Model. The scanner projects strips of white light into the body creating a raw point cloud, and the surface topography of the body is reconstructed through computer algorithms. For photonic scans, children stood in a standardized position (standing). Each child underwent two photonic scans and if the difference in waist circumference was >10 mm, a third scan was performed, and arithmetic means of each measurement were calculated. We selected 38 photonic scan measurements to be extracted from the Body Scan Model, in order to represent all body segments (that is, trunk, arms and legs). We did not extract data on measurements that served primarily for clothes design (for example, collar circumference and chin–floor height, bust-specific measures relevant to women and locations of joints and landmarks). A total of 3350 children were scanned, corresponding to 90% of the children who attended the 6-year follow-up. An example of body scan model generated by three-dimensional photonic scanner is presented in Supplementary Figure S1.

### Principal component analysis (PCA)

The analyses included 24 measures from the 38 extracted from scanner. Left-side (for example, left thigh circumference) and some specific measures (elbow circumference and chin height, for example) were not included. Independent dimensions of body shape and size were identified by PCA of these 24 measurements, including circumferences (waist, hip, seat, wrist, chest, abdomen, thigh, knee, calf, neck and biceps), lengths or widths (waist width, abdomen width, inside leg length, sagittal diameter, upper shoulder diameter and arm length) and volumes or surface areas (body volume, torso volume, arm volume and leg volume; torso surface area, leg surface area and arm surface area) plus height (from manual anthropometry). Components with eigenvalues >1 were selected (Supplementary Figure S2). We used a rule-free approach to PCA and considered as representative of each component all measurements with an absolute factor loading >0.22 or <−0.22 to find a set that optimized percentage of variance explained and interpretability (Table 2). Varimax rotation provided only marginal improvement in interpretation of components, and we therefore analysed PCA results without this approach. Component scores were calculated for each child in the sample, multiplied by 100 and standardized to provide a more convenient scale. Loadings of the second component were multiplied by −1 so that positive loadings represent central measurements and negative loadings represent those of the periphery (‘Central:peripheral ratio'). In a preliminary analysis, we ran PCA separately by sex, but as the results were similar in boys and girls, we decided to run PCA with both sexes grouped.

### Other statistical analyses

We estimated cross-sectional Pearson's correlations between the components of body shape and size and variables derived from anthropometry (weight, BMI, waist circumference, height, and waist:hip ratio) and DXA total and regional body composition (fat mass, lean mass and bone mass and trunk, android and gynoid fat mass). Analysis of variance was used to compare mean component scores according to SEP (quintiles) based on Brazilian National Wealth Index,^23^ gender (male/female), parent-reported skin colour (black/brown/white), birth weight (<2500/⩾2500 g) and BMI *z*-score by comparison to the World Health Organization 2006 reference (‘normal weight' −2 to ⩽+1 s.d./‘overweight' >+1 to ⩽+2 s.d./‘obese' >+2 s.d.).^24^ All analyses were carried out in Stata version 13.1 (Stata Corp., College Station, TX, USA).

## Results

Children's characteristics are summarized in Table 1. More than 70% were white and almost 40% were overweight or obese. Boys were taller and had higher DXA fat-free mass than girls, while girls had higher hip and thigh circumferences and higher total fat mass and trunk, android and gynoid fat mass. There were no sex differences in weight, BMI or waist circumference.

Analysis of the photonic scan measurements identified four independent components (Table 2). The first component (which we termed ‘Corpulence') showed positive loadings with the following measures: waist, hip, seat, chest, abdomen, knee, calf and biceps circumferences, sagittal diameter, waist and abdomen width, body volume, and torso volume. The second component (‘Central:peripheral ratio') showed positive loading with torso surface area (and positive trends with all other measures of central body area) and negative loadings with thigh circumference, inside leg length, arm length, leg volume and leg surface area. The third component (‘Height and arm lengths') showed positive loadings with height, arm length, torso surface area and arm surface area and negative loading with thigh circumference. Finally, the fourth component (‘Shoulder diameter') showed positive loadings with upper shoulder diameter and wrist circumference and negative loading with arm volume. After standardization, all components have a mean of 0 and an s.d. of 1.

To assess how well these four components of body shape and size were captured by traditional anthropometric and body composition measurements, we assessed their correlations (Table 3). Corpulence was strongly correlated (Pearson coefficients *r*>0.70) with weight, BMI, waist circumference, height and all DXA total and regional body composition measures. In contrast, components 2–4 showed at best only weak–moderate correlations (all *r*<0.45) with traditional anthropometric and DXA parameters. Notably, waist–hip ratio showed modest association with Corpulence (*r*=0.39) and weak association with Central:peripheral ratio (*r*=0.14). An exploratory analysis of non-traditional measurements showed that Central:peripheral ratio was positively correlated with (photonic scan measured) waist:thigh circumference ratio (*r*=0.74).

Differences in the four components by sex, birth weight, BMI z-score, SEP and skin colour are shown in Table 4. We observed that the magnitude of differences was higher to Corpulence in almost all independent variables, despite differences seen in other three components. There was no sex difference in Corpulence, but boys had higher Central:peripheral ratio, Height and arm lengths and Shoulder diameter than girls. In addition, children with low birth weight had lower *z*-score of Corpulence (−0.37) and Height and arm lengths (−0.13) but higher values for Central:peripheral ratio (0.13) and Shoulder diameter (0.15) than other children. Furthermore, obese children had substantially higher values for Corpulence and Central:peripheral ratio (presenting a *z*-score mean of 1.7 and 0.4, respectively), and lower values for Height and arm lengths and Shoulder diameter than other children (*z*-score mean of −0.3 and −0.1, respectively). Children from higher SEP showed higher *z*-score mean of Corpulence when compared with children from lowest SEP (0.3 vs −0.3). Furthermore, they also presented higher mean of Height and arm lengths, despite the magnitude of difference being lower than for Corpulence (−0.3 vs 0.1). Finally, white children had higher values for Corpulence and Central:peripheral ratio than brown or black children, while black children had higher values for Height and arm lengths (Table 4).

## Discussion

Our study, using a novel comprehensive assessment by 3-D photonic scanning, identified four independent components of children's body shape and size in a Brazilian birth cohort. The most notable finding was that traditional anthropometric and body composition measurements were strongly correlated with only one of these four components, namely Corpulence or ‘overall body size'. Therefore many traditional measurements in children, used for predicting future disease risk, seem to be limited by their assessment of the same single dimension of body shape and size and fail to capture the other three components. Moreover, despite the magnitude of differences have been higher to Corpulence in almost all independent variables, those other traditionally poorly captured components (Central:peripheral ratio, Height and arm lengths and Shoulder diameter) showed significant patterning by sex, birth weight, obesity status, SEP and skin colour, which suggests that they might contribute independently to prediction of disease risks.

Boys presented a more central body shape than girls, indicating that the typical sex-divergent pattern of adult body shape, often assumed to reflect sex hormone actions, are already seen in prepubertal children. Similarly, previous studies have reported sex differences in body shape in prepubertal children. Taylor *et al.*^3^ reported that prepubertal boys had more waist fat but less hip fat than girls, and those differences widened in pubertal stages.^3^ Another study reported that 5–7-year boys had higher waist circumference and waist–hip ratio than girls.^25^ In our cohort, unadjusted analyses showed that boys and girls had equal average waist circumference, but their mean hip circumference was 1 cm smaller than that of girls. After adjustment for height and total body composition, we observed that boys had higher waist circumference but lower hip and thigh circumferences than girls (data not shown). Hormonal differences explain the sex dimorphism in body shape after puberty^6^ but could also contribute to differences in prepubertal children. Garnett *et al.*^26^ found significant sex differences in the circulating concentrations of estradiol, leptin and testosterone before puberty.

Associations with sociodemographic indicators, BMI and birthweight tended to be much stronger for the first component than for the other three. Birth weight has been inconsistently associated with body shape and body fat distribution in childhood.^27, 28, 29^ We found that children with low birth weight presented markedly lower values for Corpulence, slightly lower values for Height and arm lengths and slightly higher Central:peripheral ratio. It indicates that in our cohort low birth weight children tend to remain generally smaller at 6 years and present a more central body shape than other children. We also observed significant differences in body shape and size by SEP and skin colour. The higher values for Corpulence in children from high SEP and white skin colour is consistent with previous reports of higher BMI and body fat mass in this cohort and in other studies^30, 31, 32^ and likely reflects differences in nutrition and other childhood factors. Nevertheless, further studies should examine whether differences in components of body shape and size are related to lifestyle factors, such as feeding habits, physical activity, sedentary behaviour and so on in childhood.

Body shape measured by 3-D photonic scanning has been reported in adults,^14, 33^ in whom differences were detected between Thai and British adults^16^ and by parity in women.^34^ To our knowledge, this is the first study to use 3-D photonic scanning to assess the variability and potential determinants of body shape in children.

3-D photonic scanning enabled us to assess different dimensions of body shape, beyond traditional anthropometric assessment. Thus we could see that traditional measures capture the same single component of children's body shape and size. Our findings suggest that some of those other, yet overlooked, components of body shape and size could be estimated by additional simple anthropometric approaches (for example, the ratio between tape-measured waist and thigh circumferences); however, future studies would be required to validate that approach and to show predictive value of the other three components.

Strengths of our study are the large sample size and the low rate of attrition owing to loss of contact or refusal (9.8%), which minimizes bias. In addition, comprehensive assessments using traditional anthropometry and DXA allowed comparability of 3-D scanning to other techniques. Limitations include the single age point at 3-D scanning; therefore, future assessments are needed to inform how applicable our findings are to other ages and to assess how body shape changes with age. Moreover, the disease relevance of our new components of body shape is yet unknown and will require future studies. Finally, 3-D photonic scanning has not been validated in Brazilian children. In a validation study in a multi-ethnic sample of children of similar age in the United Kingdom, 3-D photonic scan outcomes were found to overestimate manual measurements for almost all outcomes, varying from 0.6 cm to calf girth up to 3.7 cm to chest girth.^18^ However, ranking consistency was very high, and when compiling composite 3-D scan outcomes as in our analysis, biases in individual girths are likely to cancel out.

In conclusion, one component Corpulence explained almost 70% of the variance in children's body shape and size, and traditional anthropometry and body composition measures were strongly correlated with Corpulence but not with the other three components identified here. Differences in these three novel components of body shape and size by sex, birth weight, obesity status and skin colour suggest that they might potentially contribute additionally to future disease risks; however, this hypothesis requires testing in future studies.

## Key messages

1. Traditional anthropometric and body composition measurements capture only one single component of children's body shape and size. Through 3-D photonic scanning, it was possible to identify four distinct components.
2. Different associations between these components and sex, birth weight, SEP and skin colour might indicate their potential relevance to disease risks.
3. Sex differences in Central:peripheral ratio and other dimensions of body shape are seen even in prepubertal children, suggesting the influence of factors other than pubertal and adult sex steroid exposures.

## Figures and Tables

**Table 2:** Principal components for body shape and size in children aged 6 years: the 2004 Pelotas Birth Cohort Study

*Photonic measurement*	*Component 1 ‘Corpulence'*	*Central:peripheral ratio*	*Height and arm lengths*	*Shoulder diameter*
	*Loadings*			
Waist circumference	**0.24**	0.11	−0.03	−0.03
Hip circumference	**0.24**	0.09	−0.00	−0.04
Seat circumference	**0.24**	0.08	−0.00	−0.04
Chest circumference	**0.23**	0.08	−0.03	−0.03
Abdomen circumference	**0.23**	0.10	−0.05	−0.03
Waist width	**0.23**	0.11	0.01	−0.02
Abdomen width	**0.23**	0.10	−0.01	−0.04
Knee circumference	**0.23**	−0.04	−0.08	−0.03
Calf circumference	**0.23**	−0.00	−0.09	−0.04
Biceps circumference	**0.22**	0.03	−0.07	0.04
Sagittal diameter	**0.22**	0.10	−0.02	−0.05
Body volume	**0.24**	0.06	0.03	−0.05
Torso volume	**0.23**	0.20	0.10	−0.02
Inside leg length	0.09	**−0.62**	−0.02	0.03
Leg volume	0.21	**−0.28**	−0.19	−0.03
Leg surface area	0.19	**−0.39**	−0.19	−0.01
Thigh circumference	0.20	**−0.27**	**−0.33**	−0.03
Torso surface area	0.20	**0.24**	**0.24**	0.01
Arm length	0.08	**−0.24**	**0.66**	0.24
Height	0.17	−0.21	**0.39**	0.04
Arm surface area	0.20	−0.11	**0.30**	−0.04
Upper shoulder diameter	0.07	0.11	−0.21	**0.81**
Wrist circumference	0.12	0.03	−0.07	**0.39**
Arm volume	0.13	0.05	−0.05	**−0.29**
Neck circumference	0.18	0.10	−0.00	0.09
Variance explained	66.7%	8.2%	4.9%	4.1%

Values in bold correspond to the loadings considered as representative of each component.

**Table 1:** Description of sociodemographic and anthropometric variables of children by sex: the 2004 Pelotas Birth Cohort Study

*Categorical variables*	*Male,* N *(%)*	*Female,* N *(%)*	P*-value*^a^
*SEP quintiles*			0.427
First (upper)	388 (22.6)	372 (22.9)	
Second	351 (20.4)	367 (22.7)	
Third	383 (22.3)	362 (22.4)	
Fourth	298 (17.4)	253 (15.6)	
Fifth (lower)	297 (17.3)	266 (16.4)	
			
*Skin colour*			0.526
White	1143 (70.8)	1092 (72.2)	
Brown	219 (13.6)	185 (12.2)	
Black	252 (15.6)	236 (15.6)	
			
*BMI category*			0.409
Normal weight	1088 (65.2)	1005 (63.7)	
Overweight	289 (17.3)	302 (19.1)	
Obese	291 (17.5)	272 (17.2)	
			
*Birth weight, g*			0.036
<2500	130 (7.5)	156 (9.6)	
⩾2500	1593 (92.5)	1471 (90.4)	
*Continuous variables*	*Mean (s.d.)*	*Mean (s.d.)*	P*-value*^b^
*Anthropometry*			
Weight (kg)	25.1 (5.8)	24.9 (6.1)	0.228
Height (m)	1.22 (0.1)	1.20 (0.1)	<0.001
Body mass index (kg m^−2^)	16.9 (2.8)	17.0 (3.1)	0.190
Waist circumference (cm)^c^	58.4 (7.3)	58.4 (7.8)	0.951
Hip circumference (cm)^c^	66.9 (7.1)	67.9 (7.5)	<0.001
Thigh circumference (cm)^c^	38.3 (5.3)	38.9 (5.7)	0.002
			
*DXA variables*			
Fat mass (kg)	4.9 (3.9)	6.3 (4.2)	<0.001
Fat free mass (kg)	20.2 (2.5)	18.5 (2.4)	<0.001
Trunk fat mass (kg)	2.1 (1.9)	2.7 (2.1)	<0.001
Android fat mass (kg)	0.4 (0.4)	0.5 (0.4)	<0.001
Gynoid fat mass (kg)	1.1 (0.7)	1.3 (0.7)	<0.001

Abbreviations: BMI, body mass index; DXA, dual X-ray absorptiometry; SEP, socio-economic position.

a Chi-squared.

b Analysis of variance.

c Measured by photonic scanner.

**Table 3:** Correlations between 3DPS components in 6-year-old children and traditional anthropometric and body composition measures: the 2004 Pelotas Birth Cohort Study

*Variable*	*Component 1 (‘Corpulence')*	*Component 2 (‘Central:peripheral ratio')*	*Component 3 (‘Height and arm lengths')*	*Component 4 (‘Shoulder diameter')*
*Traditional anthropometry*				
Weight	**0.99** (**<0.001)**	0.04 (0.023)	0.03 (0.116)	−0.04 (0.052)
BMI	**0.93** (**<0.001)**	0.20 (<0.001)	−0.18 (<0.001)	−0.07 (<0.001)
Waist circumference	**0.95** (**<0.001)**	0.13 (<0.001)	−0.06 (0.002)	−0.04 (0.022)
Height	**0.70** (**<0.001)**	−0.30 (<0.001)	0.43 (<0.001)	0.04 (0.038)
Waist−Hip ratio	0.39 (<0.001)	0.14 (<0.001)	−0.09 (<0.001)	0.03 (0.185)
Sitting height	0.69 (<0.001)	−0.17 (<0.001)	0.29 (<0.001)	0.01 (0.546)
Leg length	0.48 (<0.001)	−0.32 (<0.001)	0.44 (<0.001)	0.05 (0.004)
				
*Non-traditional measures*				
Waist-thigh ratio	0.07 (<0.001)	**0.74** (**<0.001)**	0.46 (<0.001)	0.01 (0.471)
				
*Body composition (DXA)*				
Fat mass	**0.93** (**<0.001)**	0.12 (<0.001)	−0.09 (<0.001)	−0.07 (<0.001)
Lean mass	**0.78** (**<0.001)**	−0.08 (<0.001)	0.21 (<0.001)	0.03 (0.157)
Bone mass	**0.82** (**<0.001)**	−0.10 (<0.001)	0.15 (<0.001)	−0.02 (0.332)
Trunk fat mass	**0.91** (**<0.001)**	−0.15 (<0.001)	−0.10 (<0.001)	−0.06 (0.001)
Android fat mass	**0.90** (**<0.001)**	−0.17 (<0.001)	−0.11 (<0.001)	−0.06 (0.001)
Gynoid fat mass	**0.93** (**<0.001)**	−0.10 (<0.001)	−0.08 (<0.001)	−0.07 (<0.001)

Abbreviations: BMI, body mass index; DXA, dual X-ray absorptiometry; 3DPS, three-dimensional photonic scan. Pearson correlation coefficients (*P*-values) are displayed. Values in bold represent high correlations coefficients according to Pearson's correlation between the components of children's body shape and size and anthropometry and body composition variables.

**Table 4:** Sociodemographic and anthropometric factors associated with the four components of body shape and size in 6-year-old children: the 2004 Pelotas Birth Cohort Study, Pelotas, Brazil

	*Component 1 (‘Corpulence')*	*Component 2 (‘Central:peripheral ratio')*	*Component 3 (‘Height and arm lengths')*	*Component 4 (‘Shoulder diameter')*
*Gender*	0.293	<0.001	<0.001	<0.001
Male	0.02	0.07	0.09	0.08
Female	−0.02	−0.08	−0.10	−0.09
				
*Birth weight*	<0.001	0.031	0.028	0.011
<2500g	−0.37	0.13	−0.13	0.15
⩾2500g	0.04	−0.01	0.01	−0.02
				
*BMI (z-score)*	<0.001	<0.001	<0.001	0.012
–2 to ⩽+1	−0.53	−0.08	0.10	0.03
>+1 to ⩽+2	0.39	−0.07	−0.13	0.01
>+2	1.66	0.37	−0.29	−0.12
				
*SEP*	<0.001	0.075	0.004	0.137
1 (lowest)	−0.29	0.05	−0.09	0.04
2	−0.10	0.04	0.02	0.05
3	0.02	0.02	−0.06	0.02
4	0.16	−0.04	0.07	−0.04
5 (highest)	0.33	−0.10	0.11	−0.09
				
*Skin colour*	0.027	<0.001	0.033	0.859
White	0.03	0.04	−0.01	−0.01
Brown	−0.09	−0.04	−0.05	0.02
Black	−0.08	−0.19	0.13	−0.01

Abbreviations: BMI, body mass index; SEP, socio-economic position. Mean components scores and *P*-values are displayed from analysis of variance.
