# Supplementary material for: Body shape and size in 6-year old children: assessment by three-dimensional photonic scanning
Source: Int J Obes (Lond). 2016 Apr 5;40(6):1012–7. doi: 10.1038/ijo.2016.30 (PMC4899819; doi:10.1038/ijo.2016.30)
Supplement: Supplementary Figure 1 [file ijo201630x1.docx]

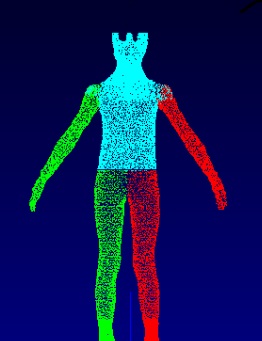

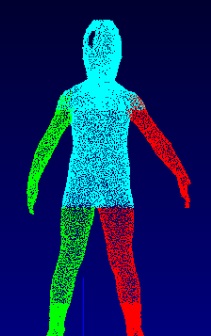


**B**

**A**

**Supplementary Figure 1:** Typical body model generated by Three-Dimensional Photonic Scanner. (A - Boy in the 50^th^ percentile of *Corpulence*; B - Girl in the 50^th^ percentile of *Corpulence*)
