# Supplementary material for: Body shape and size in 6-year old children: assessment by three-dimensional photonic scanning
Source: Int J Obes (Lond). 2016 Apr 5;40(6):1012–7. doi: 10.1038/ijo.2016.30 (PMC4899819; doi:10.1038/ijo.2016.30)
Supplement: Supplementary Figure 2 [file ijo201630x2.docx]

**Supplementary Figure 2:** Screeplot of eigenvalues from principle components analysis of body shape
